# Zooming into the binding groove of HLA molecules: which positions and which substitutions change peptide binding most?

**DOI:** 10.1007/s00251-015-0849-y

**Published:** 2015-06-04

**Authors:** Hanneke W. M. van Deutekom, Can Keşmir

**Affiliations:** Theoretical Biology and Bioinformatics, Utrecht University, Padualaan 8, 3584 CH Utrecht, The Netherlands; Department of Experimental Cardiology and Department of Clinical Epidemiology, Biostatistics and Bioinformatics, Academic Medical Center, Amsterdam, The Netherlands

**Keywords:** HLA molecules, Binding groove, Polymorphism, Peptide binding

## Abstract

Human leukocyte antigen (HLA) genes are the most polymorphic genes in the human genome. Almost all polymorphic residues are located in the peptide-binding groove, resulting in different peptide-binding preferences. Whether a single amino acid change can alter the peptide-binding repertoire of an HLA molecule has never been shown. To experimentally quantify the contribution of a single amino acid change to the peptide repertoire of even a single HLA molecule requires an immense number of HLA peptide-binding measurements. Therefore, we used an in silico method to study the effect of single mutations on the peptide repertoires. We predicted the peptide-binding repertoire of a large set of HLA molecules and used the overlap of the peptide-binding repertoires of each pair of HLA molecules that differ on a single position to measure how much single substitutions change the peptide binding. We found that the effect of a single substitution in the peptide-binding groove depends on the substituted position and the amino acids involved. The positions that alter peptide binding most are the most polymorphic ones, while those that are hardly variable among HLA molecules have the lowest effect on the peptide repertoire. Although expected, the relationship between functional divergence and polymorphism of HLA molecules has never been shown before. Additionally, we show that a single substitution in HLA-B molecules has more effect on the peptide-binding repertoire compared to that in HLA-A molecules. This provides an (alternative) explanation for the larger polymorphism of HLA-B molecules compared to HLA-A molecules.

**Electronic supplementary material:**

The online version of this article (doi:10.1007/s00251-015-0849-y) contains supplementary material, which is available to authorized users.

## Introduction

Human leukocyte antigen (HLA) molecules play a central role in induction of T cell responses, because a T cell recognizes an infected cell only in the context of peptides presented by HLA molecules. A fascinating aspect of HLA molecules has been their large polymorphism at the population level. Different HLA molecules present a different set of peptides to T cells and to NK cells, and this property has been, most likely, the main driving force behind the HLA polymorphism. Substitutions within the peptide-binding groove are expected to change the binding preference most, leading to a different peptide-binding repertoire. A novel HLA molecule with a (slightly) altered peptide-binding repertoire will be maintained in the population, if the new binding preference provides fitness advantage to its host.

Because of the high polymorphism and their role in generating an immune response, it is not surprising that HLA molecules are associated with more diseases than any other region of the genome (Horton et al. 2004; de Bakker et al. 2006). The most intriguing associations are those where one HLA molecule is associated with a disease, while a closely related HLA molecule is not. For example, HLA-B*27:05 is associated with ankylosing spondylitis, in contrast to HLA-B*27:09 (Fiorillo et al. 1998), even though the difference between these two molecules is minimal; the substitution of an aspartate with a histidine on position 116. HLA-B*42:01 is associated with a better control of HIV, while HLA-B*42:02 is not, despite the fact that these molecules differ only at position nine (Kloverpris et al. 2012). Similarly, HLA-B*35:03 is associated with a fast progression to AIDS, while HLA-B*35:01 is not; these two HLA molecules differ at position 116 only (Gao et al. 2001). In all these examples, the HLA molecules differ at a single position, yet this difference results in a completely different disease outcome. As the positions mentioned in these examples are part of the peptide-binding groove, these observations suggest that different disease associations might be explained by changes in the peptide-binding repertoire of an HLA molecule.

To investigate the impact of a single substitution on the total peptide repertoire of an HLA molecule, one needs to compare the peptide repertoire of that HLA molecule to the peptide repertoires of other HLA molecules that differ at a single position. Obviously, this demands labor-intensive experiments, such as peptide elution assays, involving large set of HLA molecules. As a consequence, the experimental binding data to address this question is limited in both the number of HLA molecules and the number of peptides per HLA molecule. Therefore, we used the in silico predictor NetMHCpan (Nielsen et al. 2007; Hoof et al. 2009) to address this question. Our results showed that the effect of a substitution on the peptide-binding repertoire depends on both the position that is substituted and the chemicophysical properties of the amino acids involved. In general, the most polymorphic positions have the highest influence on the peptide-binding repertoire, suggesting that only the substitutions that actually change the peptide-binding repertoire, and thereby the function of HLA molecules, have been selected through evolution. Finally, we show that the effect of a substitution on the peptide-binding repertoire is significantly larger in HLA-B molecules compared to HLA-A molecules, suggesting that novel HLA-B molecules could easily evolve through point mutations and, therefore, approach a larger diversity at the population level.

## Materials and methods

### Selection of HLA molecules

HLA molecules from the National Marrow Donor Program database (http://bioinformatics.nmdp.org/, HLA Haplotype Frequencies, May 2010), where a frequency is reported for at least one of the four ethnic groups, were included in this study. Only 34 polymorphic positions in the peptide-binding groove are used by NetMHCpan method to predict peptide binding. Therefore, only one of the HLA molecules that have the exact same combination of amino acids in these 34 positions is included in our analysis, resulting in 80 HLA-A and 171 HLA-B molecules. The selection was based on the frequency of the molecules; for example, among the HLA molecules with identical binding predictions, we kept the most frequent ones in our analysis.

### Experimentally verified HLA peptide-binding data

All peptides that were reported to bind to HLA molecules were downloaded from the IEDB (Vita et al. 2010) and SYFPEITHI (Rammensee et al. 1999) in March 2013. The IEDB database provides the binding affinity for each peptide, and those with an intermediate or high binding affinity were classified as binders (<500 nM). SYFPEITHI database mainly contains the ligands identified during peptide elution assays, and therefore, all the entries downloaded from SYFPEITHI database are considered as binders.

### Generating predicted peptide-binding repertoires

We used NetMHCpan to predict peptide-binding repertoires of HLA molecules (Nielsen et al. 2007). NetMHCpan is a neural network-based predictor that is trained on peptide-binding data to a set of MHC molecules, including most common HLA-A and HLA-B molecules. The most remarkable property of this method is its ability to extend predictions to MHC molecules that were not part of the training set. This is achieved by coding every MHC molecule by a subset of the amino acids presenting the binding groove of that MHC molecules. The residues used for this coding are defined as being within 4.0 Å of the peptide in any of a representative set of HLA-A and HLA-B structures (Nielsen et al. 2007). We randomly selected 10^5^ 9mer peptides from a large set of 9mers derived using all protein sequences in the Uniprot database (as used by Hoof et al. 2009). Subsequently, we used NetMHCpan version 2.4 (Hoof et al. 2009) to predict binding affinities of these 9mers for the selected HLA molecules. We defined the top 1 % of best binding peptides as the peptide-binding repertoire of a specific HLA molecule.

### Comparing peptide-binding repertoires

We used the overlap of the predicted peptide-binding repertoires of two HLA molecules as a measure for the functional similarity of the corresponding HLA molecules. The overlap is calculated as the percentage of peptides that are among top 1 % best binding peptides of both HLA molecules, i.e., the percentage of peptides that are present in both peptide-binding repertoires (see Figure S2). Our results are not depending on the 1 % threshold we have chosen: the overlaps of peptide-binding repertoire using the top 2 % are highly correlated to the overlaps using the top 1 % (*r*^2^ = 0.98, *p* = 0).

### Generating in silico HLA molecules

To generate in silico HLA molecules, we used the most common HLA molecules, i.e., those with a frequency larger than 0.5 % in one of the four ethnic groups within the National Marrow Donor Program (http://bioinformatics.nmdp.org/), as a template. An in silico HLA molecule is generated by mutating one out of 34 positions used as an input for NetMHCpan. We did not use random substitutions; instead, we replaced the amino acid with another amino acid occurring in that position in any of the HLA molecules in the training set of NetMHCpan, thereby keeping the degree of polymorphism on a specific position in the generated HLA molecules similar to naturally occurring ones. By doing so, we hoped to obtain reliable predictions for peptide binding of these in silico HLA molecules. The substitutions were locus-specific; for example, an in silico HLA-A molecule would be generated by implementing a substitution found only in HLA-A molecules. This resulted in not having any changes at ten positions in HLA-A and seven positions in HLA-B (i.e., positions 7, 24, 45, 59, 69, 84, 118, 143, 147, and 159 and 7, 59, 73, 84, 118, 150, and 159, respectively). Using all possible substitutions, we generated 3,072 HLA-A and 5,396 HLA-B molecules.

### Simpson reciprocal index

To calculate the degree of polymorphism for each position within the peptide-binding repertoire, we calculated the Simpson reciprocal index (SRI; Simpson 1949):

$$ SRI=1/\sum_{i=1}^{20}{f}_i^2 $$

where *f*_*i*_ is the fraction of amino acid *i* at that specific position. The SRI is a number between 1 and 20 and defines a weighted number for the amount of different amino acids observed on a specific position.

## Results

### Experimentally verified peptide-binding measurements suggest that single amino acid substitutions affect HLA function

**Fig. 1:**
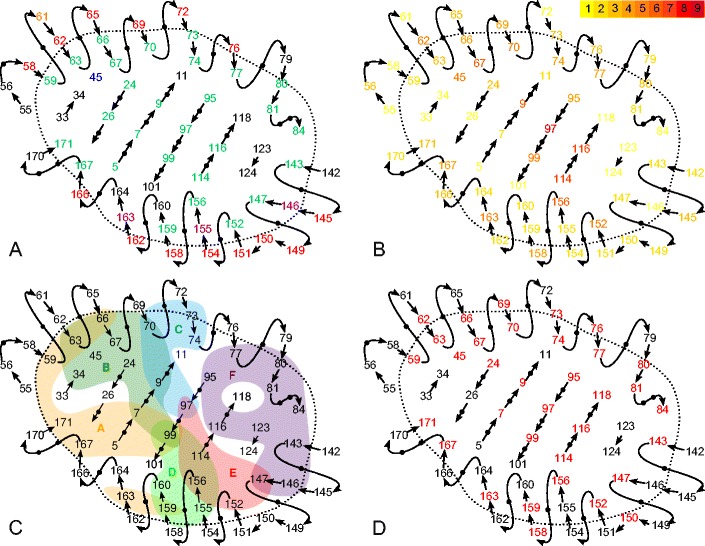
A schematic overview of the binding groove (Adapted from Garrett et al 1989. Nature, Macmillan Publishers Ltd., copyright 1989). The *straight lines in the center* indicate the platform of β strands, the upper helix is the α1 helix, and the lower one is the α2 helix. Panel **a** shows which residues can influence peptide binding and T cell recognition according to Bjorkman et al. (1987). Residues pointing toward the peptide binding site and can interact with the peptide are colored in *green*. Those pointing up from the peptide binding site and can interact with the T cell receptor are colored in *red*. Residues 146, 155, and 163 are pointing both up and into the binding site (*purple*). Residue 61 points away from the binding site (*orange*), and residue 45 is located behind the α1 helix, pointing toward it (*blue*). The remaining residues are shown in *black*. Panel **b** shows the polymorphism of every position on the α1 and α2 domains. The number of different amino acids in our HLA subset (*n* = 251; see “Materials and methods”) found at a single position is indicated in the *colored bar*, numbers are colored accordingly. Panel **c** shows pockets *A–F* as defined in HLA-A2 (Saper et al. 1991). The positions in *red* in panel **d** are used as input for the in silico predictor NetMHCpan

A randomly chosen pair of HLA molecules can be different from each other at several positions. Since it is unclear whether or not the effect of substitutions is additive, we restricted ourselves to the effect of single substitutions. Obviously, not every single substitution would result in a novel peptide-binding preference. To start with, we focused on the substitutions of the 61 positions, which were shown by Garrett et al. (1989) to be part of the peptide-binding groove (Fig. 1a). These positions cover all six peptide-binding pockets (Fig. 1c).

To analyze the effect of a single amino acid substitution in the peptide-binding groove, we compared the peptide-binding repertoires of the corresponding HLA molecules. We combined the peptide-binding data from the IEDB (Vita et al. 2010) and SYFPEITHI (Rammensee et al. 1999) databases, and found data for 26 pairs of HLA molecules that differ in only a single position among these 61 positions. Unfortunately, only for ten pairs of HLA-sufficient binding data (at least 20 peptides) are reported to reliably compare the presented peptide repertoires (Table 1). For those pairs, we calculated the percentage of peptides that bind both HLA molecules or to neither of them (Table 1). Although very limited, these data suggest that a specific single substitution can change the binding repertoire of an HLA molecule. For example, there is only 66 % overlap between the peptide-binding repertoires of HLA-A*02:01 and HLA-A*02:07, which differ at position 99 only (Table 1). Clearly, the experimental data is far from being sufficient to be able to quantify the effect of single substitutions in different residues of the peptide-binding groove, but it still demonstrates that single substitutions can affect peptide-binding drastically.

**Table 1:** The number of known binders and nonbinders for the pairs of HLA molecules that differ at one position

		Position	Total	Binders	Nonbinders	%
HLA-A*02:01	HLA-A*02:16	163	581	60	494	95.4
HLA-A*02:04	HLA-A*02:01	97	7	4	2	–
HLA-A*02:05	HLA-A*02:02	9	96	48	40	91.7
HLA-A*02:06	HLA-A*02:01	9	3,313	1,110	1,608	82
HLA-A*02:06	HLA-A*02:10	99	2	0	2	–
HLA-A*02:06	HLA-A*02:14	95	2	0	1	–
HLA-A*02:07	HLA-A*02:01	99	70	22	24	65.7
HLA-A*02:12	HLA-A*02:01	156	820	119	661	95.1
HLA-A*02:14	HLA-A*02:05	156	3	0	2	–
HLA-A*26:01	HLA-A*26:02	116	233	57	146	87.1
HLA-A*33:01	HLA-A*33:03	171	4	1	2	–
HLA-B*15:09	HLA-B*15:10	114	2	2	0	–
HLA-B*27:04	HLA-B*27:10	77	3	2	1	–
HLA-B*27:05	HLA-B*27:03	59	117	16	100	99.1
HLA-B*27:05	HLA-B*27:09	116	29	22	3	86.2
HLA-B*27:10	HLA-B*27:05	152	3	2	1	–
HLA-B*27:20	HLA-B*27:06	114	1	1	0	–
HLA-B*35:01	HLA-B*35:03	116	10	1	6	–
HLA-B*35:01	HLA-B*35:08	156	42	36	4	95.2
HLA-B*39:09	HLA-B*39:01	99	4	4	0	–
HLA-B*41:03	HLA-B*41:01	95	1	1	0	–
HLA-B*41:03	HLA-B*41:02	97	5	5	0	–
HLA-B*44:03	HLA-B*44:02	156	507	140	319	90.5
HLA-B*44:05	HLA-B*44:02	116	4	4	0	–
HLA-B*55:02	HLA-B*55:01	77	1	1	0	–
HLA-B*55:02	HLA-B*56:01	163	1	1	0	–

The total is the number of peptides for which binding data is available for both HLA molecules. The % defines the percentage of peptides that bind to both HLA molecules or to neither one of them, which is not calculated for cases where we have too few data. Note that only IEDB provides data on nonbinders

### In silico prediction methods are sufficiently sensitive to detect the differences between peptide-binding repertoires of highly similar HLA molecules

To get a better understanding of which residues and which substitutions affect the peptide binding most, we used the in silico program NetMHCpan (Nielsen et al. 2007; Hoof et al. 2009) to predict peptide repertoires of several HLA molecules. We restricted ourselves to HLA molecules for which the population frequencies are reported and for which NetMHCpan predicts a unique peptide-binding repertoire, resulting in 251 different HLA molecules that we can further analyze (see “Materials and methods”). Using this HLA set, we counted the number of different amino acids occurring in the 61 positions that is largely describing the peptide-binding groove (Fig. 1b). While some positions are conserved among the 251 HLA molecules (e.g., positions 33 and 34), others can have up to nine different amino acids (e.g., position 97). The NetMHCpan method uses a subset of the residues in the peptide-binding groove to predict the peptide binding (indicated in Fig. 1d).

The performance of the HLA peptide-binding prediction programs has been validated several times. For example, Peters et al. (2006) demonstrated that the correlation between the predicted and the measured HLA peptide-binding affinity is similar to the correlation between the affinity measurements obtained from two different labs using the same techniques. Obviously, the efficiency of the predictions differs between the HLA molecules. For our case, it is essential that NetMHCpan can reliably predict the differences in the peptide-binding repertoires of HLA molecules that are very similar. To test this, we predicted the peptide-binding repertoire of closely related HLA molecules reported in literature, which were not used to train the NetMHCpan method.

The peptides that are presented by HLA molecules originate from ER. However, it is rather hard to predict which set of peptides normally would be present in ER, as the amount of peptides depends heavily on the abundance of cellular proteins. Therefore, we used 10^5^ randomly chosen peptides from the proteins present in UniProt database as a possible set of peptides available in ER for all HLA molecules (see “Materials and methods”). For each HLA molecule in our data set, we predicted the peptide binding to these peptides using NetMHCpan. NetMHCpan produces a predicted HLA peptide binding for every HLA peptide pair that is given as input. We ranked the predictions for each HLA molecule and defined the top 1 % best predicted binders as the peptide-binding repertoire of the HLA molecule.

**Fig. 2:**
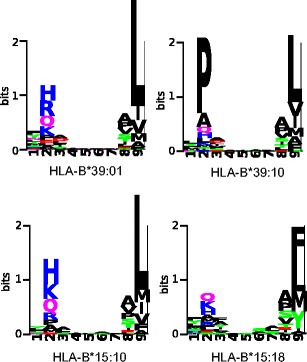
Peptide-binding motifs. For those HLA molecules discussed in the text, the top 1,000 best predicted binders out of 10^5^ natural peptides are presented in a sequence logo. These motifs are very similar to the motifs on the MHC motif viewer website (Rapin et al. 2008), where motifs from other HLA molecules are also available

Yague et al. (1998) showed that, although HLA-B*39:01 and HLA-B*39:10 differ only at position 67, HLA-B*39:10 has a more hydrophobic B pocket than its closest neighbor HLA-B*39:01. Since HLA-B*39:10 peptide-binding data was not used to train the netMHCpan method, we investigated whether the predicted peptide-binding repertoires of these two molecules reflect the reported difference. In Fig. 2, we plot the predicted binders for these two HLA molecules in the form of a sequence logo. Indeed, our predictions suggest a very dominant preference for Proline on the second anchor residue for HLA- B*39:10 in contrast to the preference for basic residues for HLA-B*39:01 (Fig. 2, upper panel). Out of the 1,000 predicted binders for HLA-B*39:10 and those for HLA-B*39:01, only 318 are present in both peptide-binding repertoires, suggesting that the binding preferences of these two HLA molecules are largely distinct.

Another example from literature is HLA-B*15:10 and HLA-B*15:18, which differ only at position 116. Prilliman et al. (1999) showed that a tyrosine to serine polymorphism at this position drastically changes the peptide-binding repertoire. Even though neither of these two molecules are present in the training set of NetMHCpan, we predict a distinct peptide-binding repertoire for these HLA molecules, with a clear difference in the preferred amino acid in the F pocket (Fig. 2, lower panel). The overlap between predicted peptide repertoires of these two molecules remains at 45 %. These examples demonstrate that NetMHCpan accurately captures differences in peptide-binding repertoires of very closely related HLA molecules and, therefore, should be a sufficient tool to quantify functional differences between HLA molecules.

### The effect of single amino acid substitutions on the peptide-binding repertoire is highly diverse

Our HLA set contains 138 pairs of HLA molecules with a single substitution. The positions that differ among these 138 pairs demonstrate which single substitutions occurred in contemporary HLA molecules, and thus, have been evolutionarily selected. Some positions, e.g., 156, 97, or 116, play a dominant role in peptide binding, as they more frequently differ among HLA pairs than the other positions (Figure S1). Moreover, Figure S1 reflects also the higher polymorphism found in HLA-B molecules compared to HLA-A molecules: we found more single substitution HLA pairs in B locus than in A locus.

**Fig. 3:**
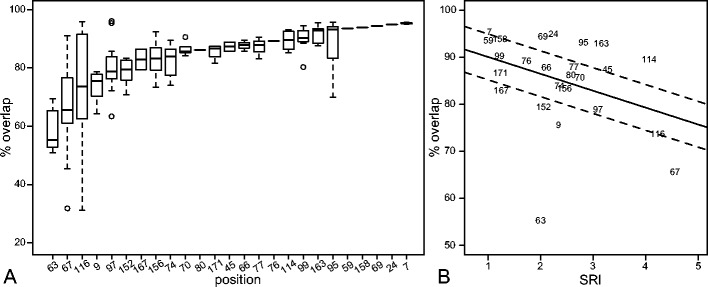
Substitutions in different positions affect the peptide binding differently. Panel **a** shows the percentage of overlap between the peptide-binding repertoires of two HLA molecules that differ at a single position in the peptide-binding groove. The positions are ordered according to the median overlap within all pairs of HLA molecules. Panel **b** shows the correlation between the median overlap of all pairs of HLA molecules differing at a particular position and the SRI of that position. The *black line* represents the linear regression, and the *dashed lines* represent the standard error. (Spearman correlation, *ρ* = −0.38, *p* = 0.06)

To see the effect of single substitutions in 138 HLA pairs, we used the overlap of the predicted peptide-binding repertoires (Figure S2) as a measure for the functional similarity of the corresponding HLA molecules. The overlap is simply defined as the ratio of number of peptides that are predicted to bind to both HLA molecules with respect to the total predicted peptide repertoire of a single HLA molecule (*n* = 1,000 peptides for each HLA molecule for the sake of simplicity). A low overlap indicates a large difference in the peptide repertoires of two HLA molecules. We found the lowest overlap, i.e., largest functional difference, for position 63 (Fig. 3a). Substitutions on positions 67, 9, and 116 also have a large impact on the peptide-binding repertoire. In contrast, changing positions 7, 24, 59, 69, or 158 hardly changed the peptide-binding repertoire of the HLA molecule (overlap > 93.5 %, Fig. 3a). Interestingly, although position 156 differed most often between pairs of HLA molecules (Figure S1), it does not drastically change the peptide repertoire (median overlap 83.3 %, Fig. 3a). This suggests that other mechanisms might drive this polymorphism. Indeed, it has been shown that differences on position 156 alter CTL recognition (Herman et al. 1999).

As mentioned earlier, the polymorphism of positions in the binding groove are highly variable (Fig. 1b). Obviously, the polymorphism per position might on its own explain the variation on peptide repertoire overlap, i.e., positions where substitutions have a larger effect on peptide binding might be more polymorphic than the positions with medium or no impact on peptide binding. To test if this is the case, we calculated the degree of polymorphism for each position in terms of the reciprocal Simpson index (SRI; Simpson 1949). The SRI gives a weighted number of different amino acids observed at a specific position within our HLA set (see “Materials and methods”). The median overlap between the peptide repertoires of HLA molecules at a position correlates significantly with the SRI of the that specific position (Fig. 3b, Spearman correlation, *ρ* = −0.38, *p* = 0.06). This result suggests that in general, the residues of the binding groove that heavily affect the peptide binding are also the residues where most polymorphism is found. To our knowledge, this is the first time that a correlation was found between the functional divergence and the polymorphism of MHC molecules, although this relationship was suggested right after the biological function of MHC molecules was discovered (Doherty and Zinkernagel 1975).

Positions 9, 63, 67, 152, and 167 are clear outliers in Fig. 3b, where a substitution has a larger effect on the peptide-binding repertoire than expected by the polymorphism at that position. Most of these positions are part of the B pocket (positions 9, 63, and 67) or the F pocket (position 116) (Fig. 1c).

**Fig. 4:**
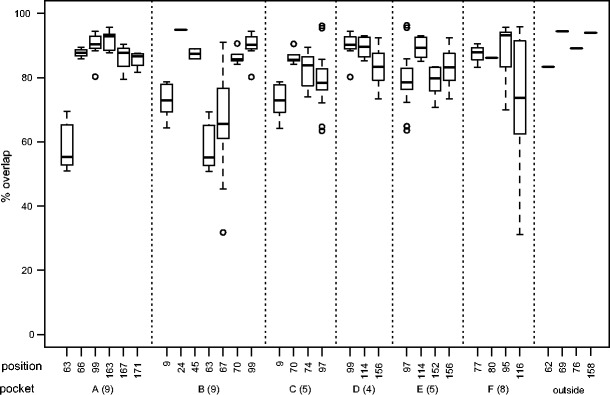
The effect of substitutions in different HLA peptide-binding pockets. The positions are ordered according to the pockets, and several positions are represented in more than one pocket (Fig. 1c). The *number in between brackets* indicates the total number of positions within the pocket that are also among the 34 positions used by netMHCpan (Fig. 1d); note that this number also includes monomorphic positions

Nevertheless, being part of the B or F pocket does not necessarily imply that changing these positions would change the peptide-binding repertoire, because positions 24, 45, 70, and 99 of the B pocket and 77, 80, and 95 of the F pocket hardly change the functionality (Fig. 4).

### Not only the position but also the nature of the amino acid substitutions affects the peptide-binding repertoire

The results presented in Figs. 3 and 4 suggest that the specific position of the substitution has a large effect on the peptide binding and, therefore, the function of the HLA molecules. However, within a position, there might be a large variance in the overlaps we predict as well (Fig. 3a). Position 116 is a perfect example to demonstrate this effect (Table 2). A few substitutions at this position lead to hardly any change in peptide binding, which can be explained by the nature of the substitutions, e.g., a phenylalanine (F) to tyrosine (Y) substitution (both large and nonpolar), or an aspartate (D) to asparagine (N) substitution (both large and polar). The largest effect on peptide-binding repertoire is found for the serine (S) to tyrosine (Y) substitution, which changes a small amino acid to a larger and more hydrophobic one. In other words, substituting an amino acid with one that has completely different physicochemical properties seems to have the largest effect on the peptide-binding repertoire. We tested this hypothesis by making use of the scores of a general amino acid substitution scoring matrix BLOSUM62 (Henikoff and Henikoff 1992), where positive scores indicate the more likely substitutions and negative scores less likely substitutions. Indeed, the percentage overlap found at position 116 (Table 2) correlates very significantly with the BLOSUM score (*p* < 0.0003, Spearman’s rank correlation), and similar results were found for positions 67 and 97 (SRI > 3, with *p* < 0.03 and *p* < 0.008, respectively, data not shown), suggesting that substitutions that are less likely to occur change the peptide-binding repertoire most. Interestingly, the effect of a substitution from a serine to a tyrosine is variable, e.g., can result in an overlap of 31 % as well as 75 % in the peptide-binding repertoire (Table 2), suggesting that the background of an HLA molecule determines largely the effect of a substitution.

**Table 2:** The overlap of peptide-binding repertoires of HLA molecules with a substitution on position 116

HLA	aa	HLA	aa	% Overlap	BLOSUM62
HLA-B*15:07	S	HLA-B*15:45	Y	31.1	−2
HLA-B*15:10	Y	HLA-B*15:18	S	45.5	−2
HLA-B*35:01	S	HLA-B*35:03	F	57.2	−2
HLA-B*44:02	D	HLA-B*44:05	Y	67.8	−3
HLA-B*40:02	Y	HLA-B*40:27	N	72.2	−2
HLA-A*68:01	D	HLA-A*68:07	H	72.3	−1
HLA-B*07:02	Y	HLA-B*07:09	S	75.1	−2
HLA-B*27:05	D	HLA-B*27:09	H	77.2	−1
HLA-A*26:01	D	HLA-A*26:02	N	90.5	1
HLA-B*39:03	F	HLA-B*39:14	Y	92.5	3
HLA-B*35:02	Y	HLA-B*35:06	F	93.2	3
HLA-A*02:01	F	HLA-A*02:60	Y	95.8	3

Positive BLOSUM62 scores indicate the more likely substitutions, while negative scores indicate less likely substitutions

### HLA-B molecules are more sensitive to substitutions

**Fig. 5:**
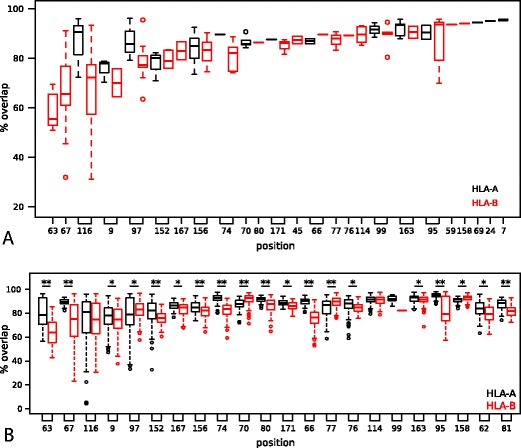
The comparison of HLA-A and HLA-B loci with respect to the sensitivity of the peptide-binding repertoire upon amino acid substitutions. Panel **a** shows the overlap between pairs of HLA molecules that naturally occur, i.e., with a known population frequency. Panel **b** shows the overlap between a naturally occurring HLA molecule (population frequency > 0.5 %, *n* = 102; see “Materials and Methods”) and an in silico generated HLA molecule that has one substitution (as described in “Materials and methods”). The *p* values are calculated using the Mann–Whitney’s *U* test with ^*^ *p* < 0.05; ^**^ *p* < 0.0001. The overall median of overlaps among HLA-B molecules is lower than that of HLA-A, paired Mann–Whitney *U* test, *p* = 0.067 for panel **a**, and *p* = 0.0027 for panel **b**

Although all HLA loci are very polymorphic, the HLA-B gene is the most polymorphic gene in the human genome (Mungall et al. 2003). One possible explanation for higher polymorphism is that amino acid substitutions effect peptide-binding repertoire much more strongly in HLA-B molecules than HLA molecules from other loci. To investigate this further, we grouped HLA pairs in our data set according to their loci and focused on the positions where substitutions are found in HLA-A (*n* = 41) and HLA-B pairs (*n* = 97). This resulted in total of 11 positions, and we compared the median percentage of overlap between HLA-A and HLA-B pairs. There is a trend suggesting that single substitutions affect the HLA-B peptide-binding repertoire more than that of their HLA-A counterparts (Fig. 5a, *p* = 0.067, Mann–Whitney *U* test). In other words, it might be easier to generate a novel peptide-binding motif through a single point mutation for an HLA-B molecule than for an HLA-A molecule. The strength of the above analysis can be improved if we could analyze all possible HLA molecules, as so far, we focused on only naturally occurring HLA molecules, i.e., the molecules where population frequencies are available. To overcome this limitation, we attempted to generate the set of all possible one amino acid substitution HLA pairs in silico as the following: we selected the most common HLA molecules (i.e., those that are found at least in 0.5 % of an American ethnic group, *n* = 102, see “Materials and methods”) and generated substitutions at each of the 34 positions of the HLA molecules that are used by NetMHCpan method to predict peptide binding. We limited the substitutions to those amino acids that are present in the set of HLA molecules used to train the NetMHCpan method, and hence, we made sure that the prediction method is trained to handle all the in silico generated substitutions (see “Materials and methods”). By using locus-specific amino acids for the substitutions, we kept the degree of polymorphism in the in silico HLA-A and HLA-B molecules similar to that in naturally occurring molecules. This procedure led to more than 3,000 in silico HLA-A and over 5,000 in silico HLA-B molecules and resulted in 22 out of 34 positions, where we can compare the effect of substitutions in HLA-A and HLA-B molecules. The peptide-binding repertoires for all in silico generated HLA molecules were predicted as described before and the overlaps between the pairs were estimated (see Fig. 5b).

The overlaps are very similar to those of naturally occurring pairs; for example, in both analysis, positions 63, 67, and 116 seem to be most crucial for peptide binding (compare Figs. 3a and 5b). A single amino acid substitution in HLA-B resulted in a significantly lower overlap in peptide-binding repertoire compared to that in HLA-A molecules in 15 out of 22 positions (68 %), suggesting that the peptide-binding groove of HLA-B molecules is more sensitive to single substitutions. Only at positions 70, 77, 97, and 158 did the substitutions result in a significantly more different peptide-binding repertoire in HLA-A molecules compared to that in HLA-B molecules. In positions 99, 114, and 116 where our analysis does not provide a significant difference, the median overlap of the peptide-binding repertoires in HLA-B pairs is smaller than that in HLA-A pairs. Taken together, our results suggest HLA-B molecules are more sensitive to point mutations.

HLA-B molecules do not only interact with cytotoxic T cells (CTL), but they are also ligands for KIR receptors on NK cells (Gumperz et al. 1997) and co-evolve with KIR molecules (Single et al. 2007). To test whether this evolution is driving the difference we observed above between HLA-A and HLA-B molecules, we grouped the HLA-B molecules into two: (i) ones carrying Bw4 motif and therefore being possible KIR ligands, and (ii) ones carrying Bw6 motif and therefore not likely KIR ligands. Surprisingly, HLA-B molecules carrying the Bw6 motifs (i.e., not being ligands of KIR molecules) are more sensitive to single substitutions compared to those with the Bw4 motif, which is significant for the individual positions 62, 70, 147, and 156 (Figure S3). These positions, however, are at distinct places within the peptide-binding groove and do not seem to be related in any way (Fig. 1c). Because the sensitivity of substitutions was not related to the Bw4 or Bw6 motifs, we conclude the difference between HLA-A and HLA-B molecules could not be explained by the additional pressure imposed on HLA-B molecules to co-evolve with KIR molecules.

## Discussion

In this paper, we investigated the impact of substitutions within the peptide-binding groove on the function of HLA molecules and found that the functional consequences of a substitution depends both on the position and on the physicochemical properties of the amino acids involved. Additionally, the effect of a single substitution also depends on the sequence of the HLA molecule, i.e., identical substitutions at identical positions can have a different effect on the peptide-binding preference (Table 2) when expressed on different HLA molecules, which show a positive correlation between the functional impact of a position in the peptide-binding groove of HLA molecules and the population diversity of that position (Fig. 3). In other words, the positions that hardly change the peptide-binding preferences are almost monomorphic at the population level. In contrast, the positions where a single substitution changes on average 20–30 % of all peptides that bind to a single HLA molecule are very polymorphic. Surprisingly, substitutions in HLA-B molecules change the peptide-binding motif more than those in HLA-A molecules, suggesting that the higher polymorphism of HLA-B molecules can be (partly) due to their higher sensitivity to point mutations.

The analysis reported in this study was only possible using the state-of-the-art in silico predictor of peptide–MHC interactions, NetMHCpan (Nielsen et al. 2007; Hoof et al. 2009), because the experimental data available is far from being sufficient to reliably study peptide-binding repertoires of such a broad range of HLA molecules. That is, using experimental data allows us to compare only at most ten pairs of HLA molecules (Table 1). The performance of MHC peptide-binding predictors in identifying T cell epitopes was demonstrated in several studies (Schellens et al. 2008; Larsen et al. 2010; Tang et al. 2011). For example, Peters et al. (2006) demonstrated that the correlation between the predicted and the measured HLA peptide-binding affinity is similar to the correlation between the affinity measurements obtained from two different labs using the same experimental techniques. Additionally, we showed in this study a few case studies where netMHCpan is able to predict subtle differences between very closely related HLA molecules. Nevertheless, HLA-C molecules were excluded from our analysis, because the predictive performance of NetMHCpan was shown to be smaller than that of HLA-A and HLA-B molecules, due to the limited amount of quantitative HLA-C binding data (Hoof et al. 2009). However, this does not necessarily reflect a shortage of our study, as HLA-C molecules play a major role as ligands for natural killer cell receptors (Blais et al. 2011), and it would be insufficient to view their evolution only in the light of their peptide-binding properties.

Our analysis focused on the effect of amino acid substitutions on peptide binding. Clearly, some substitutions can alter CTLs, change the interaction with other players of the antigen processing and presentation pathway, or change the stability of the peptide–MHC complex, and therefore affect the functional of an HLA molecule. For example, in the case of position 156 (the position where the highest number of substitutions observed among HLA molecules, see Figure S1), the peptide-binding preferences do not explain why this position became polymorphic (see Fig. 3). However, it has been shown that peptides that bind both HLA-B*44:02 and HLA-B*44:03 (which differ at position 156 only, D156L) have different confirmations, thereby affecting recognition of CTLs (Herman et al. 1999; Macdonald et al. 2003). Similar results were reported for HLA-B*35:01 and HLA-B*35:08 (L156R), suggesting that a substitution on position 156 alters CTL recognition (Herman et al. 1999; Beck et al. 1995). Another example is B*44:02 and B*44:05 which differ at position 116 only. While this position is within the F pocket, this polymorphism hardly changes the peptide binding but affects the interaction with the peptide-loading cofactor tapasin (Williams et al. 2002) and the conformational flexibility of the empty MHC proteins (Sieker et al. 2007). All these factors can be very important in shaping the functional differences between HLA molecules: if one MHC molecule is loaded with peptides within the peptide-loading complex and is dependent of the tapasin while a closely related MHC I molecule can load peptides independently of tapasin and the peptide-loading complex, the resulting peptide repertoires may differ substantially. Unfortunately, at the moment, we do not have any in silico method that we can use to address any of these important factors, and therefore, the current analysis remains bound to the effect of the substitutions on the peptide binding.

Several diseases are found to be correlated to specific HLA molecules. In an attempt to discover associations between HIV-1 and HLA molecules, a large genomewide association study was performed in a cohort of HIV-1 controllers and progressors (The International HIV Controllers Study 2010). Strikingly, SNPs in the HLA region were the only genetic components associated with successful control of HIV-1 infections. More specifically, three positions in HLA-B, 67, 70, and 97, showed the strongest association, and it was suggested that conformational differences in peptide presentation, due to different amino acids on those positions, contribute to the protective or susceptible nature of various HLA-B molecules (The International HIV Controllers Study 2010). The International HIV Controllers Study (2010) noted that position 70 is tightly coupled with positions 67 and 97. In our results, position 70 hardly changes the peptide-binding repertoire (Figs. 3a and 5b), suggesting that position 70 is “hitch-hiking” along with positions 67 and 97 in their ability to change the peptide-binding repertoire. Only position 77 was identified as an independent marker in HLA-A (The International HIV Controllers Study 2010). Also in our results, position 77 is an exception for HLA-A molecules as it changes the peptide-binding repertoire more than other positions (Fig. 5b). All in all, the large agreement between the study by The International HIV Controllers Study (2010) and ours suggests that HLA disease associations can be defined much more sensitively at the amino acid level compared to the classical HLA alleles (which, by definition, refer to the combination of amino acids).

Our results may also have clinical applications for organ transplantations. An HLA identical donor is preferred for transplantation because such a donor decreases the chance and extent of transplantation-related diseases. However, identical donors are rarely available, and subsequently, the best HLA mismatched donor should be selected. This selection is based on the mismatched locus, the number of mismatched loci, and the presence of haplotype mismatching (Petersdorf 2008). It is known that specific mismatches lead to more alloreactivity than others (Kawase et al. 2007); however, it is still unknown what the reason for these nonpermissible mismatches can be. Finding the best mismatched donor is therefore challenging. Since our results identify which substitutions are very important for changing the peptide-binding repertoire, an estimated overlap (as we performed in this study) in peptide-binding repertoire of mismatched donor and recipient could be used to optimize the HLA match. Some of the positions that came out of our analysis as the most crucial for peptide-binding preferences have previously been shown to be associated with an increased risk of transplantation-related diseases (positions 9 and 116 in HLA-A and position 116 in HLA-B; Ferrara et al. 2001; Kawase et al. 2007). Recently, Pidala et al. (2013) showed that amino acid substitutions at the peptide-binding groove increase the risk of transplantation-related diseases by focusing on positions 9, 77, 99, 116, and 156. They found a significant increase in the risk for position 9 in HLA-B and positions 99 and 116 in HLA-C, and no positions in HLA-A. Given the fact that position 9 is our second most crucial candidate for HLA-B peptide-binding preference (see Fig. 3), this result is in full agreement with our study. We believe that if position 63 was included in the study of Pidala et al. (2013), it would be also related to the high risk of transplantation-related diseases. Overall, we believe that these case studies demonstrate a possible application of our approach to compare HLA-peptide repertoires in the context of the transplantation studies. Taken together, the analysis presented here strongly suggests that the “distance” between HLA molecules at sequence level does not necessarily correlate with the differences at the functional level. Some positions in the MHC-binding groove seem to be “master” determinants of the peptide-binding specificity; for example, a single substitution at position 9, 67, 63, or 116 has, in general, a large impact on the set of presented peptides, while substitutions at several other positions barely change the peptide-binding repertoire. Our attempt to quantify these effects can have an impact in understanding HLA disease associations and clinical applications. Most importantly, we hope that our study will lead to large-scale MHC peptide-binding measurements, where our conclusions, based on the prediction methods, can be tested and revised when necessary.

## Electronic supplementary material

ESM 1 (PDF 62 kb)

ESM 2 (PDF 90 kb)

ESM 3 (PDF 82 kb)
