# Supplementary material for: Zooming into the binding groove of HLA molecules: which positions and which substitutions change peptide binding most?
Source: Immunogenetics. 2015 Jun 4;67(8):425–36. doi: 10.1007/s00251-015-0849-y (PMC4498290; doi:10.1007/s00251-015-0849-y)
Supplement: Supplementary file 1 — (PDF 62 kb) [file 251_2015_849_MOESM1_ESM.pdf]

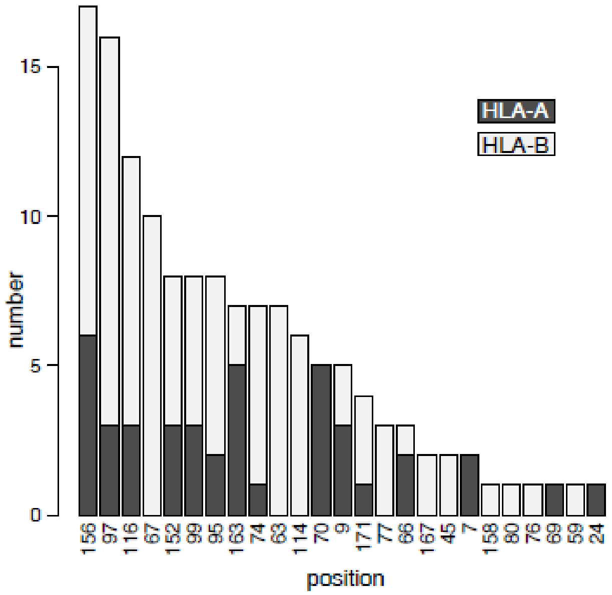

Fig. S1 The distribution of HLA pairs with a single substitution (n=138). The positions are ranked according to their frequencies among the HLA pairs.
