# Supplementary material for: Zooming into the binding groove of HLA molecules: which positions and which substitutions change peptide binding most?
Source: Immunogenetics. 2015 Jun 4;67(8):425–36. doi: 10.1007/s00251-015-0849-y (PMC4498290; doi:10.1007/s00251-015-0849-y)
Supplement: Supplementary file 2 — (PDF 90 kb) [file 251_2015_849_MOESM2_ESM.pdf]

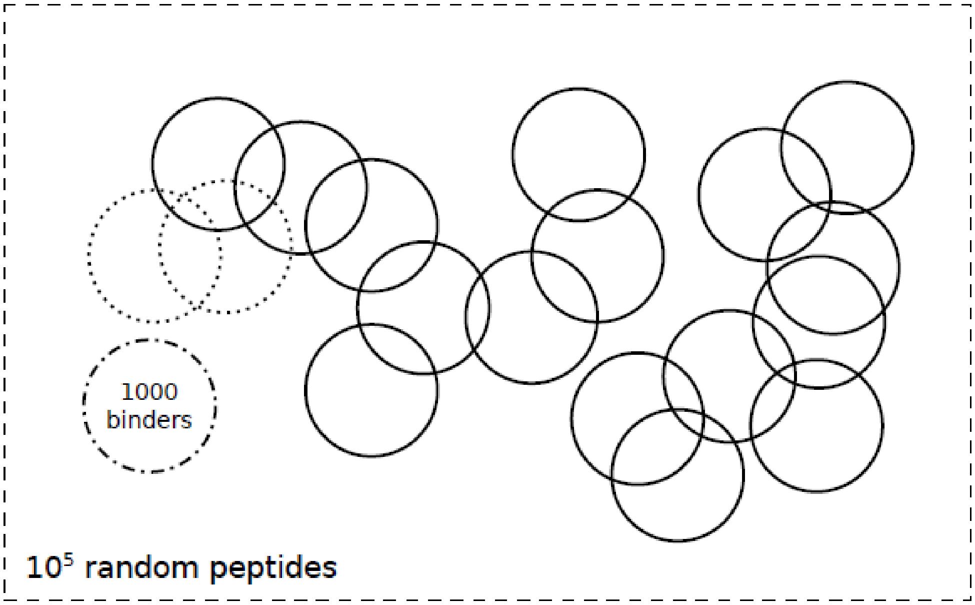

Fig. S2 A simplified representation of the estimated overlap between peptide binding repertoire of HLA molecules. Each circle represents the peptide binding repertoire of a single HLA molecule, i.e. the top 1000 best binding peptides, as shown in the dash-dotted circle. By predicting the peptide binding repertoire of all HLA molecules, we can subsequently investigate the overlap between each pair of HLA molecules by looking at their intersection.
