# Supplementary material for: Zooming into the binding groove of HLA molecules: which positions and which substitutions change peptide binding most?
Source: Immunogenetics. 2015 Jun 4;67(8):425–36. doi: 10.1007/s00251-015-0849-y (PMC4498290; doi:10.1007/s00251-015-0849-y)
Supplement: Supplementary file 3 — (PDF 82 kb) [file 251_2015_849_MOESM3_ESM.pdf]

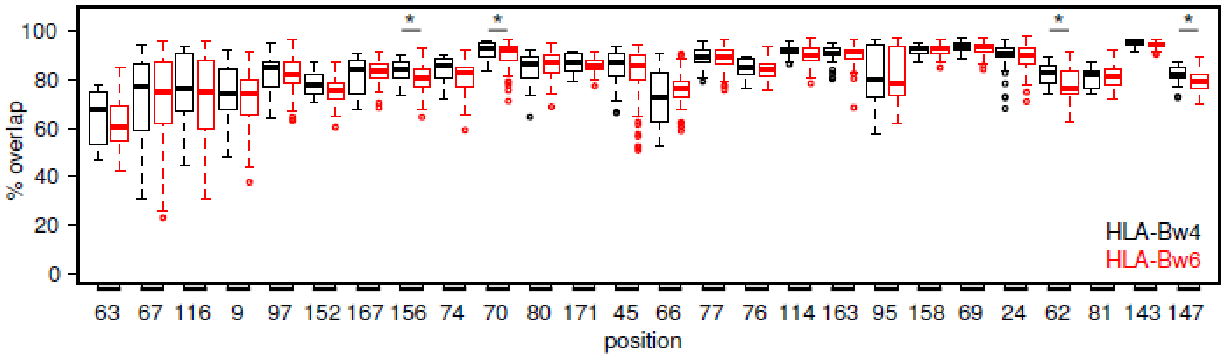

Fig. S3 The HLA-B molecules carrying BW6 motif are more sensitive to amino acid substitutions. HLA-B molecules are divided into two groups based on Bw4 and Bw6 motif. The p values are calculated using the Mann Whitney U test with * $p < 0.05$. The overal median of the HLA-B molecules with a Bw6 motif is lower than that of molecules with a Bw4 motif, paired Mann Whitney U test, $p = 0.0001$.
